# Immigrant parents' perspectives on children's oral health and barriers to a culturally adapted intervention in Norway

**DOI:** 10.3389/froh.2025.1726535

**Published:** 2026-01-06

**Authors:** Mariam Reda, Fungisai Gwanzura Ottemöller, Manal Mustafa

**Affiliations:** 1 Oral Health Centre of Expertise in Western Norway, Bergen, Norway; 2 Department of Health Promotion and Development, Faculty of Psychology, University of Bergen, Bergen, Norway; 3 Department of Global Public Health and Primary Care, University of Bergen, Bergen, Norway

**Keywords:** child, dental caries, immigrants, feeding behavior, oral hygiene, parents, preschool, qualitative research

## Abstract

**Introduction:**

A previous culturally adapted oral health intervention was deemed ineffective in improving immigrant parents' oral health-related knowledge and attitudes. This qualitative study aimed to explore the perceived barriers to benefiting from the intervention and the perspectives of immigrant parents regarding their children's oral health behaviors, and use of dental health services.

**Materials and methods:**

Forty-five immigrant parents who previously received the intervention were invited; 12 consented to participate and were interviewed using semi-structured individual interviews. The interviews were recorded and transcribed verbatim. Data was analyzed using thematic analysis.

**Results:**

The participants were all women aged 28–44, from Africa, Asia, Eastern Europe and South America. Most were college or university-educated and were employed. Four main themes were identified: *Intervention Implementation*: Parents appreciated the use of pamphlets in their native languages, but highlighted the need to receive dental information from the public health nurses at the local health centers. *Parental perspectives on children's oral hygiene habits*: Parents demonstrated high oral health knowledge and highlighted the differences in oral hygiene habits between their home countries and Norway. *Parental perspectives on children's feeding practices*: Participants noted that sugar intake is deeply rooted in cultural and social practices, making it difficult for them to control their children's sugar intake. *Dental Health Systems for Children*: Parents expressed the need for more frequent follow-ups starting earlier than 3 years of age. They also emphasized the importance of cultural and linguistic competence and sensitivity during dental appointments.

**Conclusions:**

This study highlights the influence of immigrant parents' cultural backgrounds, daily challenges, and interactions with the Norwegian dental health system on their children's oral health practices and perceptions of the oral health intervention. The findings suggest that oral health interventions should be comprehensive, easy to remember, and delivered by credible professionals. Furthermore, to promote positive behavioral change, it is advisable to provide continuous support, repeat oral health care guidance, provide clear communication, and utilize native languages.

**Clinical Trial Registration:**

ClinicalTrials.gov Identifier: [NCT05758454].

## 1 Introduction

Early childhood dental caries (ECC), affecting the primary teeth of children under 6 years of age, remains one of the most common chronic diseases affecting young children globally (1). ECC has significant implications on children's overall health and development, such as pain, infection, difficulty eating and speaking, missing school and reduced academic performance (2–5). Evidence suggests that ECC is more common among immigrant children than children of host populations, and this may be an indicator of underlying socioeconomic disparities (6, 7). This is because ECC prevalence is strongly linked to factors such as low income, high sugar consumption, inadequate oral hygiene, and lower parental awareness of oral health practices (8, 9). The immigrant^1^ and the Norwegian-born to immigrant parents populations^2^ have grown rapidly and constitute 21.4% of the entire Norwegian population (7). In Norway, children receive free dental care, except orthodontic treatment, through the Norwegian public health system until the age of eighteen (10). Local public dental clinics schedule regular check-ups for children, typically starting at age three. If a child experiences dental issues before that age, parents consult the public health nurse or contact the dental clinic directly (10). Despite this comprehensive system, a study conducted in Oslo highlighted that preschool children with immigrant backgrounds are disproportionately affected by ECC compared to native Norwegian children (6). Additionally, parental background, knowledge, beliefs, and practices are pivotal in shaping children's oral health behaviors and outcomes, especially in early childhood, when children are dependent on their caregivers (11, 12). Several factors among immigrant families, such as cultural norms, health literacy, and compliance with dental visits, may influence how oral health is prioritized and managed at home (8, 13, 14).

Previous qualitative research in Western countries on immigrant and refugee parents' views of their children's caries experiences indicated that some carers, from various ethnic backgrounds, believed that dental visits were only necessary when problems occurred and that personal oral care was sufficient to avoid preventive care (15). Moreover, other studies reported that immigrant parents expressed little concern about their children's deciduous teeth, as they would eventually fall out anyway (15, 16). A study in the Netherlands found that children of Dutch parents from both low and high socioeconomic backgrounds, as well as immigrant parents, experienced dental caries (17). The parents in this study attributed dental decay to factors beyond their control, such as chance, genetics, or childhood health issues, rather than poor oral hygiene (17). These findings underscore the need to investigate how such beliefs may influence the uptake and effectiveness of oral health interventions. This is especially important as interventions aimed at improving health behaviors and outcomes among immigrant populations often face challenges in achieving desired results due to cultural and social factors (8, 18).

A culturally adapted intervention was conducted in Bergen, Norway, to improve immigrant parents' oral health-related knowledge and attitudes regarding their children's oral health and consequently reduce the risk of ECC among the children. The intervention was implemented twice, when the children were newborns and 6 months after. It included a 30-minute motivational interviewing session, followed by a practical demonstration of toothbrushing techniques. The participants then received a pamphlet with the same information as in the session, translated into their mother tongue (15 different languages). More details of the intervention can be found in a previous publication by Reda et al. (19). Despite its comprehensive approach, the intervention was ineffective in changing parental oral health-related knowledge, attitudes and indulgence towards their children (19).

The quantitative findings on the intervention's overall impact were valuable; however, they did not fully explain the reasons behind its lack of effectiveness. Thus, this qualitative study was conducted as part of the project to better understand the factors influencing intervention effectiveness. Qualitative research provides an opportunity to investigate the complexities of human behavior and the cultural and social influences that shape decision-making processes (20). A deeper understanding of the experiences, perceptions, and contextual factors that may have influenced participants' engagement with the intervention is crucial for improving the design of future interventions for this population.

There is a paucity of in-depth qualitative research exploring how immigrant parents understand and navigate their children's oral health needs (17). To our knowledge, this is the first qualitative study conducted in Norway to explore the perspectives of immigrant parents on their children's oral health, dietary habits, oral hygiene practices and use of dental services, as well as the perceived barriers to benefiting from a culturally adapted oral health intervention.

## 2 Materials and methods

### 2.1 Theoretical framework

The framework adopted in this qualitative study was the Theoretical Framework of Acceptability (TFA) (21). This framework was designed to assess the acceptability of healthcare interventions. It consists of seven constructs related to the intervention: affective attitude—participants' feelings about taking part in the intervention; burden—the time, financial resources, and cognitive effort required from the participants; perceived effectiveness—whether the intervention achieves its desired aims; ethicality—the intervention's congruence with the participants' values; intervention coherence—the participants' understanding of the intervention's purpose; opportunity costs—what the participant must give up to take part; and self-efficacy—participants' confidence that they can do what is required by the intervention (21).

The interview guide (Supplementary File S1) was developed based on the TFA constructs. However, the opportunity costs construct was not included, as the intervention was conducted at the health centers during the mandatory waiting time required after the children's vaccinations, so the parents did not have to give up anything to participate. We also included additional questions to explore cultural and contextual influences on the intervention's effectiveness.

### 2.2 Study design, recruitment and participants

Recruitment and interviews were conducted from May 2024 to November 2024. Parents who had participated in the intervention were initially contacted via telephone, during which MR explained the purpose of the study and invited them to participate. The interview appointments were subsequently confirmed through SMS. The interviews were conducted either in-person at the Oral Health Centre of Expertise's office in Bergen or remotely via video conferencing using Microsoft Teams, depending on the participants' preference. They were audio- and video-recorded (online interviews only). Participants who had in-person interviews were served refreshments. The duration of the interviews was 30–45 min.

Parents were recruited using purposive random sampling. First, inclusion criteria were defined as non-Western immigrant parents of children from the intervention group who received the culturally adapted intervention during the first part of the study, conducted from 2018 to 2022, when the children were newborns (19, 22). From this group, participants were then randomly selected to ensure that every eligible parent had an equal chance of being included in the study. Randomization was performed by MR through assigning each participant a random number, placing the numbers in a bowl, and selecting them at random. Parents were from non-Western regions, including Eastern Europe, Asia, Africa, Turkey, and South and Central America. The children's ages at the time of this study ranged from 5 to 7 years old.

Forty-five parents were contacted, of whom fourteen declined to participate, twelve agreed, and nineteen did not respond. Non-participation in the study was primarily due to a lack of interest or time constraints. Recruitment continued until data saturation was reached, which was determined when no new themes emerged during coding, indicating that no additional new information could be obtained (23). All participants were mothers of the children, as they had been more involved in the intervention since it began during their children's infancy and breastfeeding period.

### 2.3 Data collection and procedures

The data were collected using semi-structured open-ended individual interviews. The interviews were conducted in English, Norwegian, Arabic, and other languages, and a translator was provided where necessary (one interview was conducted in Spanish and one in an Asian Language). The interview guide was pilot tested with one participant who had participated in the first part of the study, and the questions were further edited and simplified.

The questions included were designed to initiate a discussion with the parents about the intervention (e.g., recall of the intervention, its benefits, opinions on intervention pamphlets), the children's dietary habits (e.g., indulgence of sugar intake, sugar intake frequency, routine diet at home and kindergarten), and children's oral hygiene practices (e.g., brushing and fluoride usage). Additionally, the parents discussed the differences in dental health services and cultural oral health practices between Norway and their home countries. Examples of questions were:

“The intervention sessions were provided twice only, what do you think about this?”, “Can you tell us about any challenges/difficulties you face in providing or trying to give your child healthy foods?”, and “What do you think about the oral health services for children in Norway and your home country? Are there other ways that can be helpful for your child's oral health needs?”.

The interview transcripts were anonymized, and participants were assigned pseudonyms. All audio files were transcribed verbatim after each interview. As part of the member checking procedure to strengthen credibility and transparency, interview transcripts were sent to participants for review and feedback. All interviews were translated into English to ensure consistency in the analysis.

The interviews were conducted by MR and MM, who are multilingual and proficient in Arabic, English, and Norwegian. MB is a native Norwegian speaker and a research coordinator at the Oral Health Centre of Expertise in Western Norway. She was involved in transcribing interviews conducted in Norwegian. DBL is a Spanish native speaker who is a postdoctoral researcher at the Oral Health Centre of Expertise in Western Norway and was responsible for conducting, transcribing and translating the interview in Spanish.

### 2.4 Data analysis

To ensure the quality and transparency of reporting, the Consolidated Criteria for Reporting Qualitative Research (COREQ) checklist was utilized (24). Thematic analysis, as described by Braun and Clark, was employed to identify the main themes and subthemes within the data (25). Inductive and deductive thematic analysis were employed to code, group, and interpret the data, and to generate themes using NVivo (Version 14) computer-assisted qualitative data analysis software, which was applied to the transcripts.

MR performed the initial coding, and MM, MR, and FGO had frequent meetings to discuss the codes and themes produced until consensus was achieved. First, open coding was conducted by reading the transcripts and assigning codes line by line, thereby forming the initial coding scheme. Second, the related codes were sorted and clustered to identify themes. Finally, the transcripts and analysis were reviewed, common themes and differences were integrated, and the findings were compiled.

### 2.5 Positionality

MR (MSc) is a dentist from Egypt, a native Arabic speaker, and a research fellow at the Oral Health Center of Expertise in Western Norway. Previously, she worked as a clinician and is an immigrant to Norway. MM (PhD) is a Swedish/Sudanese dentist, a native Arabic speaker, and a senior researcher who played a key role in conducting the intervention for parents participating in the study's first phase.

FGO (PhD), a Zimbabwean immigrant living in Norway, is an associate professor in health promotion at the University of Bergen. She has extensive experience in qualitative research and has conducted health behavior and education-related research. Her current research explores the settlement experiences of immigrants in Norway, focusing on both individual and structural factors. All three authors employed reflexivity, continuously reflecting on how their own identities and experiences as immigrants might influence their interactions with participants (who were also immigrants) and the interpretation of the data.

### 2.6 Ethical considerations

Participation was voluntary, with the right to withdraw at any time, and participants gave informed consent for their involvement in the study. The participants received written information about their rights in the research and how the data would be handled. As the immigrant population in Bergen is relatively small, data were tagged by pseudonyms, and identifiable information was removed from the transcripts to preserve the anonymity of the participants. Data were stored securely on a password-protected server, and access to it was restricted to the researchers involved in the project. The project is funded by the Research Council of Norway (Grant number: 331782) and the Oral Health Centre of Expertise in Bergen, Western Norway and has received ethical approval from REK-The Regional Ethical Committee (2015/27639/REK Vest). Norwegian Agency for Shared Services in Education and Research has given approval for personal data management (SIKT Reference number 778825).

## 3 Results

All 12 participants were women, aged between 28 and 44 years old. Around half of the participants had one child, and most of the children were girls. The general characteristics of the sample are presented in Table 1.

**Table 1:** Characteristics of the study participants.^a^

Participant pseudonym	Duration of residence in Norway, years	Geographical background	Education level	Employed
Mei	5	Southeast Asia	University	Yes
Olga	5	East Europe	High School	Yes
Bella	11	East Europe	University	Yes
Stephanie	3	North Africa	University	No
Riya	11	South Asia	University	Yes
Elena	26	East Europe	University	Yes
Siti	2	East Asia	University	No
Katarina	6	East Europe	University	Yes
Marianne	14	West Africa	High School	Yes
Imani	6	North Africa	University	No
Maria	8	South America	University	Yes
Lucia	3	South America	University	No

a This data was collected at the follow-up during the first part of the study (2020–2022).

Four key themes were identified during the thematic analysis: (1) Intervention implementation and parental perceptions, (2) Parental perspectives on their children's oral hygiene practices, (3) Parental perspectives on their children's dietary habits, (4) Dental health services for children.

### 3.1 Intervention implementation and parental perceptions

This theme focuses on the practical aspects of implementing the intervention, including accessibility, as well as the parents' perspectives on behavioral change. Participants' recall of the intervention content varied, with some remembering the information delivered well and others only a little. The way parents perceived the intervention content varied depending on their previous knowledge and personal experiences. Some found the information valuable and relevant:

“Yes, that I should help the children brush their teeth until they are 12 years old. I didn’t know that. I thought maybe they could brush themselves… That, I think, is important because I have a nine-year-old. So, I'll continue helping her with brushing.” (Bella)

Other parents felt the intervention was repetitive, questioned it, or disagreed with the content. *Katarina* questioned the specific advice provided during the intervention, particularly regarding nighttime breastfeeding. The information given was to avoid breastfeeding during the night when the child is 1 year or older:

“I recall something that we discussed about the breastfeeding, I guess that was in the first session prior to three years ago, that I was breastfeeding and that the milk can affect the teeth as well somehow, and I challenged this belief because I think that milk like the mother's milk could not affect the baby's teeth even though you are nursing your kid during the night, the milk goes directly in, like it does not touch the teeth throughout the breastfeeding stage.”

When participants were asked about the frequency of the intervention and whether they felt they needed additional sessions, most could not recall the exact number of sessions they had received. Some stated that they had only spoken to the researchers once, despite the intervention being administered twice. However, most participants noted that the number of sessions was sufficient. Some participants reported that the intervention did not introduce any new information, as they had been previously exposed to dental information when caring for their older children or from other sources:

“Yes, it is enough with one time. I do not think we need to meet you more times, right? Because I have experience now with brushing the teeth of the child and such. And then you teach them to brush the teeth. So, it says a lot in the brochure and… On media and such, how we shall keep the teeth clean then.” (Mei)

On the other hand, *Stephanie* believed that regular follow-up and support, along with possibly more frequent sessions, were necessary to ensure the advice given was followed, as parents tended to forget it over time. When asked whether they preferred the project researchers or the public health nurses at the local health centers to administer the intervention, some participants felt it made no difference who delivered the intervention. However, one participant felt the intervention was less effective because the information was not presented by personnel at the health center. She believed that if the information had come from health nurses, it might have been perceived as more credible and significant. Other participants also stressed the importance of receiving dental health information from the public health nurses at the health centers:

“Well, I guess because a large number (of participants) knew … that this information was a study. And it was not as if we were sitting with the health nurses receiving this information. One might think that if it were important, the health nurse would have said it herself… If I go to the family doctor and not hear this information from him, I might consider this to be theories or research, or so, not more than this.” (Stephanie)

Participants had mixed responses to the educational pamphlets they had received during the intervention. Some could not recall receiving the pamphlets at all. When the participants were asked about the use of the pamphlet, some stated that they might have skimmed through it after receiving it, but did not use it later, as it did not include new information. While others remembered some information and found it useful:

“I read it back then, but I haven’t used it since… I think it helped me become more aware of what I should do and what needs to be done to maintain good dental health. So, it helped. But maybe it could have been digital…” (Bella)

The pamphlet was delivered to participants in 15 different languages to ensure that they received it in their native language. Most participants stated that their understanding and participation in the intervention were significantly influenced by the language used in the pamphlet. Additionally, they explained that it was crucial for them to receive the intervention pamphlet in their mother tongue, particularly when they first arrived in Norway, as this improved their comprehension and lessened their anxiety. For instance, *Imani* preferred using her mother tongue in any health care setting to avoid misunderstandings, even though she understands Norwegian:

“… if you talk in your mother tongue, you understand better than if you talk in another language…in your native language, you don't need to search for the words, it's easier to understand. It's also crucial that people know their rights, especially in healthcare, including dental care.”

Given that the intervention was deemed ineffective, parents were asked what they thought could support positive changes in their oral health behaviors. Some participants explained that intrinsic motivation and external reminders are important to change negative oral health behaviors.

“It (behaviour change) has to come from within, I guess. But I mean, it helps to have these external reminders. And I see that at health centres when there are pictures of how much sugar there is in a Coca-Cola bottle, for example, or you see these visuals and you're reminded about that, and it stays with you for a little bit at least.” (Riya)

*Elena* explained that it is a matter of parental choice and awareness:

“… It's a choice parents must make… one thing is information, knowing it. Another thing is being aware and practicing it in everyday life.”

Moreover, several parents noted that their own personal negative experiences could impact their willingness to change their behavior:

“I struggled myself, I had a lot of caries when I was young, and so therefore I put a lot of attention to my kid's … oral health.” (Katarina)

### 3.2 Parental perspectives on their children's oral hygiene practices

This theme highlighted parental beliefs about their children's toothbrushing frequency and supervision, challenges in maintaining routines, and the extent to which they felt responsible or confident in guiding their children's oral health behaviors.

#### 3.2.1 Children's oral hygiene habits

Most parents stated that their children brush their teeth twice a day, with some parents reporting brushing after every sweet snack. A number of parents reported that their children enjoyed brushing and did not mind the taste of toothpaste, whereas others struggled to get their children to brush their teeth. Many parents noted that oral hygiene practices in Norway, such as brushing twice a day, were more consistent and integrated into daily routines compared to their home countries, where it was less common to brush twice daily.

“For example, in (Eastern European country), nobody brushed the teeth to a baby. When he's so young, everybody is like: Oh, why you do this? So, it was helpful (the intervention), because in our culture it's a bit strange to brush the teeth to a baby” (Olga)

Conversely, multiple parents noted that their home countries placed more emphasis on brushing than Norway.

“I believe that in our Latin American countries, they give more emphasis to oral hygiene… That is, to the children. I remember when I was little, or in the advertisements, they taught us toothbrushing before sleeping, (and) when we woke up. In the schools, they gave us the tablet for the teeth…” (Lucia)

Several parents reported using electric and manual toothbrushes interchangeably; one parent noted that an electric toothbrush made brushing more enjoyable for their child, as it made them excited to choose its design. Several parents reported giving their children fluoride tablets daily; however, some encountered challenges, as their children were reluctant to take them, and the parents were unwilling to administer them forcefully. Many incorporated daily flossing into their children's routines, but others stated that their children disliked it, fearing they might hurt themselves. Most parents also reported using fluoride toothpaste for their children. *Stephanie*, however, expressed concerns about the safety of fluoride, in contrast with the recommendation given at the intervention.

“My idea is that fluoride, no matter its percentage, causes nerve damage… So, I don't like to use this fluoride. I would buy other toothpastes. They exist, you know, toothpaste made with herbs and so. It does the same thing. And it is working with me, I use it.”

#### 3.2.2 Promoting good brushing habits in children

Parents described themselves as central figures in shaping and maintaining their children's oral hygiene routines. Many emphasized the importance of modelling behaviors, setting reminders, establishing daily brushing and strict consistent routines to instill the habit effectively. A number of parents reported introducing toothbrushing as early as when the child's first tooth erupts to form early brushing habits. The parents also described various strategies to support building brushing habits, including videos, assistance during brushing, singing songs to distract the child, reading books about dental care, warning them that their teeth would fall out or rot if they did not brush, and even using their own dental struggles as cautionary examples:

“And I believe it has something to do with routines; when the kids have been trained in this for a long time, even if they are a bit reluctant …  And they are starting to become more and more interested in their own bodies and what happens in their mouths… And we talk a lot about how you have milk teeth, and then you have permanent teeth. And now they've started to come out, and they are extra important to take care of.” (Elena)

*Katarina* emphasized the importance of not simply dictating that brushing is necessary to the child, but instead explaining why it is important, which helps children understand the reasons behind oral hygiene to foster intrinsic motivation and long-term habits:

“I think it's important to give information to the child what can happen and not just say like, oh, it's important to brush your teeth. Yes, it's important to brush your teeth, but why, why, why we brush?”

Several parents discussed the importance of encouraging their children to brush their own teeth, thereby establishing early habits of responsibility and self-care. Most parents acknowledged the need for supervision at younger ages; however, they gradually encouraged independence as their children grew older. A number of parents, however, reported that their children frequently resisted brushing independently and often requested that the parents do it for them. Modelling positive behavior, such as brushing and flossing regularly, was seen as a powerful tool in reinforcing these routines. Several parents also involved older siblings in the process, turning oral care into a shared family activity:

“I think it was good for him to see because I show him every time, you must go like this with the toothbrush, and you have to make like this… And of course, I brush him also. So, yeah, it's important to see how he has to do it.” (Olga)

One participant explained that dividing the parenting roles could help, and that immigrants usually struggle compared to native Norwegians, who tend to divide household tasks more:

“The main point is that it is the family's role to follow up on their child… until they establish the habit. Sometimes, for someone who has many children, it can be difficult. For me I have 2 children, and there is a lot of effort is done to control… But the Norwegians have it easier, they have one or two children, and the father is responsible for something and the mother for the other, so it might be easier, like it would be my job to check on the brushing at night, …. But for foreigners, most of the tasks are the mother's tasks, right?… So, the social conditions also have an effect on this topic.” (Stephanie)

#### 3.2.3 Use of alternative medicine in oral health

The use of alternative medicine and natural remedies in oral health care in their home countries was mentioned by several parents:

“It was not very common to brush twice a day… and fluoride toothpaste, that is very new to me, to kind of keep the teeth covered in fluoride and that protects us through the night… Never knew about that before I came to Norway. But I mean, (in) (East Asian country) … there are so many other ways of taking care of the teeth like herbal remedies, and you use certain plants.” (Riya)

Other participants also reported using herbal remedies in their home countries to treat dental conditions due to financial constraints:

“For instance, if there's tooth pain in (African country), they might suggest using clove instead of going to the dentist, because people might not have the money for a dental visit, so we treat problems using traditional remedies instead of going to the doctors…. they use the traditional ways more than the medical ones.” (Imani)

#### 3.2.4 Parental perspectives on caries development

When parents were asked about the importance of primary teeth, some demonstrated an understanding that they are equally important as permanent teeth in avoiding future problems. Many parents shared their perspectives on the causes of dental caries in children. Their experiences varied; some postulated that their children had caries, while others did not. Those without caries often acknowledged that it was because of their good oral hygiene habits, despite consuming sweets. While some demonstrated an understanding of factors such as sugar consumption and poor oral hygiene, others attributed caries to factors like genetics. *Bella* reported a negative encounter during her child's dental appointment, where she felt judged and blamed for her children's dental status. She attributed their dental issues to genetic factors and nutrition rather than personal caregiving:

“I said, ‘Why did this also happen to my son, who, in a way, had cavities?’ And she (the dental professional) started by saying that maybe I'm brushing badly, and that's why it happens, since all the kids have it…without saying that it could actually be genetic… But she started saying that maybe I'm a bad parent. And I didn't like that at all… I started to wonder why, when we brush so well now?…Maybe it's because he doesn't drink enough milk…Or maybe it's genetic?”

### 3.3 Parental perspectives on their children's dietary habits

Parental perceptions and management of their children's dietary habits, both in Norway and in their home countries, explores their beliefs and attitudes. It highlights parents' challenges in promoting healthy eating, limiting sugar intake, and adapting oral health practices within different cultural, social, and familial contexts. Environmental and societal factors may also influence participants' food choices and, consequently, their children's oral health. Participants discussed how peer influence, broader societal norms, the colder climate and longer winters in Norway impacted their dietary habits. Some participants noted that the weather in Norway, particularly during hot periods, contributes to increased sugar intake among children. They explained how their previous experiences shaped their current practices and described the difficulties and adjustments involved in maintaining their children's oral health after relocating to Norway. Some highlighted that traditional diets in their home countries include a higher consumption of sugar:

“In (African country), everything we consume has sugar; we can't drink without adding sugar. Children might often take a lot of sweets as well. There's a noticeable craving for sweets more so than here in Norway.” (Imani)

Conversely, others felt that the Norwegian diet was worse, particularly in terms of fast food, processed foods, and high sugar intake, which might lead to a higher caries experience in Norway compared to their home countries:

“But sugary things are so much more common in Norway than they ever were in (East Asian country), or maybe when I was growing up. So, my teeth were just fine, even though I never went to a dentist. So, I never got anything (until) way into my adulthood. So, I feel like my teeth were more taken care of in (East Asian country) than here. Fast food was very little, almost none, and sugary drinks were not available or part of the meal. I think … the eating culture was very different and much better suited for having good teeth. Whereas now the eating culture is such that… you have to put in a lot of effort to keep your teeth healthy while living in such an affluent country.” (Riya)

Many parents acknowledged that their children's food preferences had an active role in shaping their diets. While some children requested healthier options, others preferred sugary snacks and processed foods, which made it challenging for their parents to manage their diets. Several parents also expressed that their children imitated their diet. They described a range of reactions from their children when sweet treats were limited or denied. While some children accepted the refusal after explanation, many reacted with disappointment, throwing tantrums or attempting to negotiate:

“I explain to him that he cannot (have sweets) that day…. So, he sometimes gets angry and says: bad mother. I say, my love, good mother because I am teaching you, so you won't get sick when you are older… and then he gets angry for a while, and I hug him. I tell him, come let's play or watch TV… before, he used to cry as if it was the end of the world. But now not anymore, he gets angry for a little moment, and it goes away.” (Lucia)

#### 3.3.1 Sugar intake as a social practice

Several participants highlighted the influence of social norms, describing sugar consumption not just as a dietary choice but as a deeply embedded social and cultural practice. Although the parents aimed to restrict sweets to once a week, many of them struggled with their children's continuous exposure to sweets and sugar at social gatherings and family celebrations. Additionally, they expressed concerns about peer pressure and the normalization of sugar intake in everyday life. A number of parents also admitted to using treats as rewards when the children behaved well or as part of social rituals. As a result, regulating sugar consumption becomes difficult. Parents reported feeling a tension between promoting healthy habits and allowing their children to participate in social activities:

“The biggest challenge is when I have guests at home. When there are other children, or when we visit others or are at a party with other families. You see that the other children are eating candy, drinking soda, and so on. It's almost unavoidable for them to take candy or soda.” (Mei)

In Norway, it is a common tradition for children to receive sweets only on Saturdays, known as the “Saturday treat” (*Lørdagsgodt*). Parents in this study expressed varying opinions on this practice. Some viewed it as a helpful way to limit sugar intake by setting clear boundaries. Other participants sensed that it could promote excessive consumption on that single day and did not feel the need to be as strict or follow this practice. *Imani* expressed confusion about whether to follow this tradition or not:

“But I'm not sure about these candies or sweets on Saturdays. Are they good or not? I don't know. I've heard some people say that deprivation can make a child more eager for the thing they're denied. In my opinion, I shouldn't deprive the child. Because when they grow up, they'll crave it more and might consume it excessively, especially if they've been deprived of things like (brand of soda).”

#### 3.3.2 Nutrition at home and kindergarten

Children's nutrition at home is often shaped by culture, family preferences, such as vegetarianism, and health needs. The parents reported that most of their children eat healthy diets, but some children were also allowed sugary snacks. Several parents struggled to get their children to eat vegetables, while others reported using smoothies or freshly squeezed juices. In kindergarten, multiple parents appreciated that their children had regular meals including fruits, vegetables, and healthy foods. However, others expressed dissatisfaction with the types of food provided, raising concerns about the prevalence of sugar and processed foods, especially during birthday celebrations. Additionally, most parents reported that they gave their children packed lunches from home.

A few parents reported that they do not communicate with schools regarding the food provided for their children, and consequently, they feel a lack of control over this aspect. However, one parent noted that she participated in discussions with the kindergarten about the food allowed there. A number of parents mentioned that kindergartens do not address dental care, but one parent highlighted that her children learned about dental caries at the kindergarten through the story of “Karius and Baktus.” Regarding dietary improvements at the kindergarten, *Imani* suggested introducing healthier food options:

“Maybe the diet here, especially at schools and in the kindergarten. There could be a better focus on the food they offer, like for it to be adjusted to the weather. In the school system, food seems to be more fixed around certain types like bread, white cheese and brown cheese. You are obligated to eat this food. Like having a warm meal once a week is not enough, in my opinion. They take care of the economics of it and so, yes I agree, but you say that children are the most important thing. So, you have to provide them with good food and good things.”

#### 3.3.3 Parental strategies to encourage healthy eating

Parents reported several strategies to limit their children's sugar intake. For example, restricting access to sweets by controlling their availability at home and offering non-sweet alternatives. Additionally, several parents taught their children about the effects of sugar on health, promoting moderation and teaching them to balance sweets with healthy foods. When shopping, parents distracted or removed their children from aisles at stores that contain candy to avoid cravings:

“You can talk with the child in that way that she feels safe and secure and understands the subject that… that's not healthy for you. The healthy option is an apple, or sometimes you need to distract as well… if the situation, in the shop, let's say, she sees the candy and she wants it… Maybe we need to leave the shop, leave the aisle where the candies are” (Katarina)

Parents reported a variety of snacking habits among their children, including adopting a habit of taking snacks directly after meals. A few parents diluted juice with water to reduce the amount of sugar, while others opted for homemade cookies or healthy snacks. Negotiation on the timing, quantity and type of sweets was also a strategy that was used:

“Conflict in chocolate, yes of course… If she insists, I try to tell her that okay, at the end of the week she would not get this candy… sometimes she is responsive, and sometimes she accepts the deal as in ‘I would eat chocolate now and I don’t want this candy bought at the end of the week.” (Stephanie)

Additionally, one parent noted that detecting hunger cues in children helps her design better nutritional strategies:

“I usually notice that children like sugar because they come from school hungry, so if I make sure that on the way home, she would eat her lunchbox, her appetite becomes less, or her asking for sugar becomes less.” (Stephanie)

Elena discussed the importance of consistency, and she pointed out that sugar intake is a global issue:

“So being consistent and saying, ‘Okay, this is dinner; you can either eat it, and if you're not hungry or don't want to eat it, you can get up from the table and leave.’ And then when they get really, really hungry, they sit down and eat…I think generally in the world, chocolate, soda, all sweets, and ice cream have become so easily accessible that it's hard to say no. It's so easy to go to the store and buy chocolate or something. Chips, for that matter, are no better.”

### 3.4 Dental health services for children

Parents' experiences and perceptions of the dental health services for children in Norway highlights their views on access to services, the quality of care provided, communication with dental professionals, and how the systems support preventive practices. They shared their experiences and observations regarding their children's dental appointments, sharing both positive aspects and areas needing improvement. Few parents reported receiving advice and guidance on healthy behaviors to adopt from their dentists, while others did not. Most parents reported that communication with dental service personnel typically occurs in Norwegian, but that the dentists were flexible in translating information to English if needed.

“I have some problems with the language. I might use English sometimes when I don't understand Norwegian. So, the dentist explains it in English, but he also uses pictures and explains to me like that… If I meet a dentist who is from my home country, it's much easier for me to ask questions.” (Mei)

The use of translators was reported to be a critical factor in facilitating communication between parents and dental professionals. While several parents appreciated the support, others expressed concerns about accuracy, availability, confidentiality and the translator's understanding of medical terminology:

“Yes, I would have liked that (having a translator) because I had just arrived. I practically didn't understand anything. So, I think that it is important, yes, to have someone that explains to you in your own language, no?” (Lucia)

*Stephanie* was not so positive:

“No, this translator thing is a failing matter… because the families don't like to talk in front of him. And sometimes he would also use a dialect that is different, right?… you have 11% who are immigrants, so you should have the same percentage (of immigrant health workers) distributed in the health system.”

Parents reflected on their children's both positive encounters and areas of dissatisfaction during dental visits, sharing a range of experiences from initial fear to enjoyment. Most parents reported that their children had positive experiences and that small gifts given to their children during dental visits helped reduce anxiety and reinforced positive behavior. Several parents expressed that it is important for dentists to communicate directly with the children during dental visits. They believed that when dentists engage children in conversation, explain procedures in a child-friendly manner, and respond to their questions, it helps build trust and reduces fear:

“It was important to communicate with the child, because sometimes the child would listen to orders and would think that mom doesn't want to do this or so, but when they listen to it from them (the dentists) for a couple of times, the child understands that this is a real thing.” (Stephanie)

Most parents stated that they trust the dental health services for their children, and that they will get the necessary help if needed:

“We can just contact a dentist if we need to. So, we always have access to resources, so it's not a big problem for me. So, I'm satisfied.” (Siti)

Parents' feedback on how the Norwegian dental health services could better serve immigrant families focused on enhancing communication, accessibility, and cultural understanding within dental services. Most participants explained that their children visit the dentist when they receive appointments for dental checks, or when the child has complaints, or for specialist treatment, and most expressed satisfaction with this frequency. However, some participants preferred more follow-ups. Additionally, some expressed that the follow-up system should start earlier than 3 years:

“I told my husband, but in (South American country) they take the babies earlier to dental check, and he told me no, that we have to wait a while, a year, I don't know, I think it should start early with the oral health topic in children” (Lucia)

A number of parents, however, mentioned that they were not always contacted for routine check-ups, and they had to take the initiative to schedule appointments themselves.

“… it was a bit surprising that we didn't get called in this year. I was the one who booked the appointment this time… because she did not have any invitation.” (Maria)

Some participants emphasized the importance of repetition of oral health advice, particularly for immigrant families:

“The problem with immigrants is that these topics (oral health topics) are not on their minds to begin with, as I told you. So, they themselves, before the children, need to be followed up… so, there should be reminders every time about what they have done, if they have reduced the sugar quantity, if they brush or not, and to check on the children's teeth.” (Stephanie)

Several participants reported that their children's dental appointments were occasionally rushed and expressed a desire for more time with the dentist. Others indicated a need for greater flexibility in selecting their child's dental care provider. Additionally, few parents noted prolonged waiting times for appointments and expressed a preference for more timely access to dental services:

“But often, you get an appointment much later. For example, we come in and they look and say, ‘Yes, we need to fix this, but it can wait.’ … You end up waiting 3–4 months. But it's not like that for adults who go to the dentist. I know it's completely different. If I have something like a grade 2 or grade 3 issue, I have to get it done within a short time, not wait 4 months. And baby teeth deteriorate much faster.” (Bella)

## 4 Discussion

This study aimed to explore the perspectives of immigrant parents regarding their children's oral health behaviors and dietary habits. It also aimed to understand the perceived barriers experienced by parents in adopting healthy oral behaviors for their children after participating in a culturally adapted oral health intervention. Four main themes emerged, capturing the various challenges, the sociocultural and structural factors that immigrant parents usually encounter in terms of their children's oral health.

### 4.1 The intervention: parental reflections on implementation and impact

According to the Theoretical Framework of Acceptability (TFA) (21), the ability to remember the intervention reflects the cognitive effort required from participants, which reflects the *burden* associated with memory. Research has shown that recall of intervention content is a challenge in health-related interventions, which can impact their effectiveness (26). This was evident in our study, as a few parents expressed limited recall of the intervention and the need for follow-up via phone calls or regular contact with health personnel/research team. This highlights the importance of maintaining engagement over time and aligns with TFA's concepts of affective attitude and self-efficacy, where ongoing support can improve motivation and confidence in sustaining behavior change. Previous research indicates that interventions that include repeated sessions and reminders have demonstrated better long-term effectiveness (27).

One participant expressed that receiving oral health information from researchers, rather than from routine health professionals like public health nurses, may have negatively affected the perceived credibility of the intervention. This relates to TFA's *ethicality*, which reflects the alignment of the intervention with the participants' belief system. This suggests that implementation strategies should involve healthcare facilities and personnel to reinforce key messages and build interaction. Norwegian studies on public health nurses and oral health present conflicting findings. A study by Skeie et al. concluded that oral health is generally a minor component of the educational curriculum for public health nurses and is not considered a priority (28). However, another study reported that public health nurses at the health centers were aware that an immigrant background was a risk factor for ECC and were involved in identifying and referring immigrant children to dentists (29). The participants in our study requested a greater role for public health nurses in disseminating oral health information and supporting immigrant families.

A narrow biomedical focus often overlooks the influence of cultural practices and alternative health traditions, which continue to shape behaviors and health decisions even in new contexts. This was evident in the participants' varied *perceived effectiveness* of the intervention. Some found the information useful and new, while others considered it repetitive or conflicting with their existing knowledge. The guidance in the intervention was to brush the children's teeth twice a day using fluoride toothpaste. However, several parents spoke of using herbal remedies in their home countries to deal with oral health conditions or to brush their teeth, which research shows is a common practice among some immigrant groups (30, 31). These practices were not included in the intervention, so it may not have adequately addressed the specific cultural differences and misconceptions that some immigrant parents held.

Materials provided as supplements to offer additional information during interventions have been shown to be valuable in enhancing recall and supporting sustainable behavior change (32). Educational pamphlets were distributed during the intervention, and participants highly valued their availability in native languages, highlighting the role of language accessibility in supporting *intervention coherence* and reducing *burden*. Moreover, studies have shown that the use of linguistically adapted printed or visual materials can have a significant impact, particularly among immigrant populations that may face language barriers in healthcare settings (33, 34).

Motivation is an essential component of sustainable behavior change (35). Consistent with previous studies and participants' reports on what triggers behavioral change, our findings suggest that intrinsic motivation is a critical determinant, which highlights the importance of self-efficacy. Additionally, external reminders can also support the maintenance of behavioral change (36, 37). Moreover, in line with a study by Hilton et al., several of our participants suggested that behavior change may only occur in response to negative personal experiences, such as parents' own experience of tooth loss (15). These insights underscore the importance of interventions that not only provide information but also incorporate affective engagement, reinforcement, and self-efficacy, as well as external reminders, to promote positive oral health behaviors.

### 4.2 Navigating good oral hygiene and dietary habits among immigrant families

Oral hygiene practices and dietary habits are crucial for maintaining good oral health, especially for children. Many parents compared oral hygiene and dietary habits in Norway with those in their home countries. Some found the twice-daily toothbrushing routine in Norway to be new but beneficial. The observation that infant toothbrushing was uncommon in their home countries is consistent with the findings from a study by Riggs et al. on immigrant parents in Australia (30). Others expressed that their home countries had better oral health prevention practices and programs than Norway. As reported in another study by Arora et al., cultural background played a key role in shaping oral health behaviors, such as nutrition (38), with several parents being less strict in limiting sugary snacks, which may reflect practices in their home countries. While some noted high sugar consumption in their native diets, others pointed out that Norwegian diets included more processed foods, sweets, and fast food.

Consistent with findings from another study conducted in Australia by Riggs et al., where immigrant parents reported that caries was not a significant problem in their home countries, some of our participants expressed similar views (30). They voiced concerns that Norwegian dietary culture and norms may not support good oral health and felt that children in Norway experience more dental caries than in their countries of origin, possibly due to the high availability of foods with added sugar. Overall, parents' dietary attitudes and practices regarding their children's oral health behaviors seem to be influenced by a complex mix of cultural norms, environmental factors, social expectations, and their experiences both in Norway and their countries of origin.

Similar to the findings reported by Nogueira et al., most participants demonstrated a high level of motivation and knowledge about the importance of primary dentition and oral hygiene practices (39). One parent, however, raised concerns about the toxicity of fluoride. This was also reported in another study by Naidu et al. in Trinidad, where mothers opted for non-fluoride or herbal toothpaste due to concerns about the safety of using fluoride for their children (40). This highlights the significance of explaining the role of fluoride in protecting against dental caries and addressing parents' awareness and understanding of its importance.

Several factors may contribute to higher sugar intake or reduced oral health care in children, such as family dynamics. Although the parents expressed awareness of the potential impact of a diet high in sugar on caries development, they reported facing several challenges in preventing their children from consuming a cariogenic diet. In line with other studies, parents expressed awareness of their roles to foster good oral health habits and employed various strategies to promote healthy oral hygiene and dietary habits, including modelling, reminders, distraction, routines, negotiation, environmental control, restricting access to sweets, educational conversations, and turning brushing into a family activity (39, 41). This finding is consistent with other studies, suggesting that immigrant parents may not lack oral health-promoting knowledge; instead, other factors might influence their decision-making (42, 43). For example, one parent expressed frustration with the limited division of home labor in many immigrant households, unlike in typical Norwegian households, where tasks are shared more evenly. This finding is supported by other studies, in which mothers reported a lack of support from their husbands, which contributed to their inability to balance their time, meet the demands of daily life, and maintain good oral hygiene practices for their children (40, 44). Additionally, some also believed that factors like genetics could significantly influence caries development. This finding aligns with other studies among immigrants, where genetics is often attributed as a dominant factor in determining caries (17, 42).

Managing children's resistance to oral hygiene and their strong preference for sweets posed notable challenges for parents in establishing healthy oral health routines. Children's resistance to oral hygiene measures, especially when they are younger, and their food preferences in shaping their diets, were also prominent (17, 39, 43). As evident in another study by Arora et al., parents used sugar as a means of rewarding their children when they behaved well, which might reinforce unhealthy dietary behaviors (38). Additionally, a few parents reported that their children often request sweets or resist healthy options. This was mainly influenced by peer pressure or external exposure at social events at home and in kindergarten. Consequently, parents reported experiencing a diminished sense of control (39, 40, 45). This was primarily driven by the normalization of sugar consumption as a social and cultural practice. Parents described how sugary foods were not just dietary items but were embedded in social events, traditions, and peer interactions. Even well-intentioned strategies, such as Norway's “Saturday treat” (Lørdagsgodt), were perceived with hesitation and doubt. A number of parents thought it could encourage binge eating, while others saw it as a useful way to practice moderation. Furthermore, a study by Suprabha et al. highlighted the significant influence schools have on children's food choices (45). Kindergartens in Norway typically prohibit sugary snacks, except on special occasions such as birthdays. However, some parents called for policy changes, integrating oral health education and practices into kindergartens, as they felt they could not control their children's sugar intake while they were there (31).

### 4.3 Bridging the gap between the dental health services and immigrant families

Behavioral change extends beyond individual choices and actions; it is also shaped by the systems, structures, and environments (46). Despite Norway's universal and free dental services for children, our findings revealed a lack of orientation and system-level communication, particularly among newly arrived immigrants unfamiliar with the Norwegian dental health services. This is a common finding among immigrant populations, where recently arrived immigrants often struggle to navigate the new healthcare systems, citing a need for additional assistance and clarification in understanding the unfamiliar dental system (34, 39, 41).

A few parents also reported that their countries of origin had more frequent dental check-ups for children. The dental health services in Norway, on the other hand, are characterized by relatively less frequent dental follow-ups for young children, which is adapted for the general population, given the lower prevalence of ECC (12). One must take into consideration that immigrant parents might forgo routine dental visits for their children and tend to seek care only when a dental complaint arises (13, 34, 43). This suggests a need for more targeted, preventive and health-promoting approaches among immigrants (6, 8). In our study, several parents proposed practical improvements for the dental health services in Norway. These included more frequent follow-ups, earlier dental visits (before the age of three), increased flexibility in choosing the dentist, and reduced waiting times for treatment. This finding is consistent with a previous study in the US by Cortes et al., which also identified increased waiting times as a barrier to access (41).

Language and communication emerged as central determinants of parental satisfaction and engagement. While some parents praised the efforts of dental professionals to communicate in English or use visual aids, others expressed concerns about relying on translators, particularly regarding dialects, confidentiality, and accuracy, which is a common concern for many immigrants (47, 48). Consistent with other studies, these accounts illustrate how linguistic barriers can become a significant challenge during sensitive health interactions (34, 39, 49). Additionally, past negative experiences with dental professionals, such as feeling judged or blamed for their child's dental caries, may impact trust and utilization of oral health services. Cultural competence and sensitivity when working with immigrants is crucial for encouraging engagement, building trust, improving communication, and enhancing confidence in one's ability to provide the guidance needed (50). In line with other research, parents also emphasized the importance of the dentist-child relationship. They appreciated it when dental professionals communicated directly with children, used age-appropriate language, and offered positive reinforcement through small rewards (40).

### 4.4 Strengths and limitations

The strengths of this study include its use of a qualitative approach, which enabled the in-depth examination of oral health practices among young immigrant children, a topic that has not been explored previously in Norway. Additionally, the researchers involved in the study were immigrants with diverse backgrounds, which may have helped the parents feel more comfortable expressing themselves without fear of judgment. This diversity also guaranteed that the findings were interpreted with cultural sensitivity. Efforts were made to include an equal number of parents from the various non-Western origins (Asia, Africa, Eastern Europe, South America). This approach was designed to capture the diverse perspectives of various immigrant communities, as immigrants are a heterogeneous group in terms of culture, language, and socioeconomic status (51, 52).

This study, however, has limitations. The participants may have self-reported more favorable oral health behaviors in response to the interviewers being dentists. Moreover, as these parents agreed to participate, unlike others, this may indicate that they are a more resourceful self-selected sample with more proactive oral health behaviors. As our study was conducted nearly 3 years after the intervention, this may have hindered the participants' ability to recall details. It was not feasible to conduct all the interviews in the participants' mother tongue, which might have reduced their ability to express their thoughts more freely. Most of the participants were highly educated mothers, which means the experiences of less educated mothers are underrepresented in the study. Finally, all participants were mothers, and we therefore did not capture fathers’ perspectives on their children's oral health behaviors.

### 4.5 Conclusions

This study emphasizes how immigrant parents' cultural backgrounds, daily challenges, and experiences with the Norwegian dental health services affect their children's oral health practices and their perceptions on oral health interventions. The findings indicate that oral health interventions should be comprehensive, easy to recall, and delivered by trusted professionals whom immigrants regularly encounter. Additionally, to encourage behavior change, it is recommended to offer continuous support, repeat oral health care advice, communicate clearly, and provide information in parents' native languages.

### 4.6 Future directions

Future interventions should actively involve immigrant parents in co-designing the intervention content and use trusted health professionals who can provide ongoing support on a regular basis. Policy efforts are also needed to adapt oral health services to better address the diverse needs of immigrant communities.

## Data Availability

The datasets presented in this article are not readily available because they contain information that could potentially identify participants and/or health facilities. Requests to access the datasets should be directed to mariam.reda.abdallah@vlfk.no.

## Footnotes

^1^In this paper, the term ‘immigrant’ is used to refer broadly to individuals and families with a migration background, while recognizing that this is not a homogeneous group and encompasses diverse cultural, social, and economic contexts.

^2^In Norway, second-generation immigrants who are born in Norway to immigrant parents are typically referred to as “Norwegian-born to immigrant parents”.
