# Supplementary material for: Immigrant parents' perspectives on children's oral health and barriers to a culturally adapted intervention in Norway
Source: Front Oral Health. 2026 Jan 6;6:1726535. doi: 10.3389/froh.2025.1726535 (PMC12816272; doi:10.3389/froh.2025.1726535)
Supplement: Supplementary file 1 [file Table1.docx]

Supplementary Material

**Interview Guide Questions based on the Theoretical Framework of Acceptability (TFA)**

**Perceived effectiveness-** *likelihood of the intervention aim being achieved*

1) You have previously received information explaining how to take care of your child’s teeth, provided by dental health personnel.

- What do you think about this information?
- What are the key messages you got from the intervention?
- 1. What do you remember from the intervention?
- Can you please illustrate with some examples from this intervention?

2) The intervention sessions were provided twice only. What do you think about this?

- Do you think more repetition would be more beneficial/useful?
- What do you think about receiving this intervention during every health clinic visits for a routine check-up for your child?
- How often do you think receiving the oral health information would be more useful?

**Affective attitude—***how one feels about participating in the intervention,* *and* **Intervention coherence—***understanding the intervention and how it works*

3) What did you think of the information you received during the intervention? If yes, how has it been beneficial/ how has it been helpful

- If not, in what ways do you think it can be improved?

4) Do you feel this intervention has influenced the way you take care of your child’s oral health?

- Are there things you know now about how to prevent your child from getting cavities that you didn’t know before?

5) Have you used the simplified leaflets that were provided to you during the project? How?

**Self-efficacy—***confidence in performing the required actions, and* **ethicality-** *fit with personal beliefs*

6) Can you tell us the daily meals/drinks your child receives?

- At home
- At kindergarten

7) What types of foods and drinks do you think are healthy for your child’s oral health?

- Can you tell us about any challenges/difficulties you face in providing or trying to give your child healthy foods?

8) What types of foods and drinks do you think are not good for your child’s teeth?

- Can you tell us about any challenges you face in avoiding giving your child these foods or drinks?

9) Can you tell us about your family’s daily oral hygiene routines, (brushing, washing mouth...etc.)?

10) Do you have any difficulty brushing your child’s teeth?

- If yes, can you please explain these difficulties?

11) Can you compare between the dental health system in Norway and in your homeland?

12) What was your experience in applying the oral health advice given during the intervention?

13) Your child gets free dental treatment from ages 0 till 18 years old, and receives routine checkup appointments, how often do you take your child to the dentist?

14) What do you think about the oral health services for children in Norway? Are there other ways that can be helpful for your child’s oral health needs?

**Comments: Do you have anything more you’d like to share with me?**
